# Detection of canine distemper virus in wildlife in Italy (2022–2024)

**DOI:** 10.3389/fvets.2025.1527550

**Published:** 2025-03-25

**Authors:** Flora Alfano, Maria Gabriella Lucibelli, Nicola D’Alessio, Clementina Auriemma, Simona Rea, Giovanni Sgroi, Maria Stella Lucente, Francesco Pellegrini, Georgia Diakoudi, Esterina De Carlo, Nicola Decaro, Gianvito Lanave, Vito Martella, Giovanna Fusco

**Affiliations:** ^1^Istituto Zooprofilattico Sperimentale del Mezzogiorno, Portici, Italy; ^2^Dipartimento di Medicina Veterinaria, Università degli Studi di Bari, Valenzano, Italy; ^3^University of Veterinary Medicine, Budapest, Hungary

**Keywords:** canine distemper virus, wildlife, badger, passive surveillance, Italy, phylogenetic analysis

## Abstract

CDV has been detected in a wide range of domestic and wild animal also in Italy and it is highly prone to cross-species transmission, therefore representing a significant health risk. In this study the presence of CDV and other coinfecting selected viruses, in wild carnivorans of the family Mustelidae and Canidae and rodents of the family Hystricidae, collected in Southern Italy (Campania region), in 2022–2024, was investigated. Over a period of 3 years (2022–2024), tissue samples from 136 wild animals including stone martens, porcupines, otters, wolves, martens, badgers and foxes were examined. CDV RNA was detected in 14 (10.3%) animals encompassing badgers (*n* = 6), foxes (*n* = 5), wolves (*n* = 2), and marten (*n* = 1). The complete genome of a CDV strain was reconstructed from a spleen sample of a badger. On sequence and phylogenetic analyses, the novel CDV strain belonged to the Arctic clade, which has already been reported from badger and dog in Italy. Our study contributes to extend the knowledge on the epidemiology of CDV in wildlife and confirm the need for a continuous surveillance in wild animals to monitor the circulation in wildlife of viruses pathogenic for domestic carnivores and endangered wild species.

## Introduction

Canine distemper (CD) is a highly contagious multi-systemic disease caused by canine distemper virus (CDV). The clinical disease can be sub-clinical to fatal, spreading through the bloodstream to multiple organs, with clinical signs that include respiratory distress, anxiety, conjunctivitis, anorexia, diarrhea, lymphopaenia, encephalitis, coughing, rhinorrhoea, ocular discharge and fever (1, 2).

CDV has been detected in a wide range of domestic and wild animal hosts worldwide including canids, felids, mustelids, procyonids, urisds and hyaenids (3–5). It is highly prone to cross-species transmission between domestic and wildlife reservoir hosts, therefore representing a significant conservational and animal health risk around the globe (6, 7). CDV transmission primarily occurs via various body fluids, such as respiratory droplets, ocular and nasal discharge, saliva, urine and feces (8). In Italy, CDV has been reported in both domestic and free-ranging canids, as well as in mustelids. The virus poses a significant threat to wildlife conservation and domestic animal health, making its study crucial for disease management and control. CDV is an enveloped virus with a non-segmented, negative sense, single-stranded RNA genome and belongs to the family Paramyxoviridae, genus *Morbillivirus* (3). His genome encodes six structural proteins: nucleocapsid (N), matrix (M), fusion (F), hemagglutinin (H), polymerase (L), and phosphoprotein (P) (9). Based on H-gene sequences, CDV strains have been classified into 19 genetic lineages: America-1, America-2, North America-3, South America/North America-4, America-5 (formerly regarded as a sub-genotype of America-2), Asia-1, Asia- 2, Asia-3, Asia-4, Asia-5, Europe Wildlife, Arctic, Africa-1, Africa-2, Europe-1/South America-1, South America-2, South America-3, Rockborn-like, and Asia-6 (5, 10). Recently, Lanszki et al. (11) proposed a distinct CDV classification in 16 genetic lineages based on full-genome sequences. In Italy, the presence of CDV has been reported within the Canidae family, including domestic dogs (12) and free-ranging canids, like foxes and wolves (13, 14), and also within the family Mustelidae (15–17).

In recent decades, three CDV genetic lineages have been reported to circulate in Italy: the Europe/South America-1, EuropeWildlife, and Arctic-like lineages (10). The first to be detected in both domestic dogs and wild carnivores was the Europe/South America-1 lineage, while the Europe Wildlife have been reported in wildlife and only sporadically in domestic dogs (13, 18). Conversely, Arctic-like lineage has been reported mainly in Italian dogs and, less frequently, in wildlife (14, 16, 19). These data indicate the circulation of the three different CDV strains across the domestic/wildlife interface in Italy.

This study aimed at investigating the presence of CDV and other selected viruses (rabies virus, canine coronavirus, canine adenoviruses, canine herpesvirus type 1, rotavirus, protoparvovirus carnivoran 1) from internal organs of wild carnivorans of the family Mustelidae and Canidae and rodents of the family Hystricidae, collected in Southern Italy (Campania region) in 2022–2024CDV was consistently detected in this study and genetic characterization of the identified CDV strains was performed.

## Methods

### Sample collection

A total of 1,088 tissue samples (lungs, livers, hearts, spleens, kidneys, intestines, brains and muscles) were collected from 136 wild animals including 4 stone martens (*Martes foina*), 12 porcupines (*Erethizon dorsatum*), 6 otters (*Lutra lutra*), 11 wolves (*Canis lupus italicus*), 3 martens (*Martes martes*), 34 badgers (*Meles meles*), and 66 foxes (*Vulpes vulpes*).

Samples were collected over a period of 3 years (2022–2024) at the Istituto Zooprofilattico Sperimentale del Mezzogiorno (IZSM), a ministerial institution that operates within the National Health Service. All the tested animals were found dead in the Campania region during passive surveillance and samples were collected at necropsy and were tested in this study in the framework within the diagnostic activity of IZSM. For each animal, information on gender, age, place, and date of collection were recorded. Subjects were classified as juveniles (6–12 months of age), subadults (between 12 and 24 months) and adults (>24 months).

### Nucleic acids extraction

The collected samples were processed just after their arrive in laboratory or, stored for a short time at −20°C, or for long time at −80°C, before NAs extraction. After NAs extraction the samples were immediately analyzed. The samples were subjected to NAs extraction by means of an automatic extractor (QIAsymphony, Qiagen, Hilden, Germany), using the “Virus/Pathogen” kit (Qiagen) according to the manufacturer’s protocol. The DNA and RNA quality were monitored using an exogenous Internal Positive Control (IPC) added to each sample to supervise the presence of potential PCR and RT-PCR inhibitors (VetMAX^™^ Xeno^™^ Internal Positive Controls and Assays for PCR and RT-PCR, Thermo Fisher Scientific). A sample with nuclease-free water instead of homogenate was used as a negative process control (NPC).

### Molecular screening for CDV

Identification of CDV was performed using a reverse transcription (RT) protocol followed by a quantitative real-time PCR (qPCR) assay (20).

### Molecular screening for other pathogens

All the samples were tested for rabies virus for monitoring (21). In addition, the extracts were also analyzed for the following viruses, to evaluate the presence of co-infections: canine coronavirus (CCoV) (22–24), canine adenoviruses (CAdVs) type 1 and 2 (25), canine herpesvirus type 1 (CaHV-1) (26), rotavirus A (RVA) (27), protoparvovirus carnivoran 1 (28, 29). For the latter, a qPCR able to discriminate between field variants and the vaccine virus was performed (30).

### Full-length genome sequencing of CDV

The complete genome sequencing of CDV strains identified in this study was obtained through an amplicon-based sequencing method performed according to the protocol described by Lanszki et al. (11). PCR products were pooled in equimolar ratios, quantified by Qubit dsDNA HS assay (Thermo Fisher Scientific, Waltham, MA) and used for library preparation and adapter-ligation by Ligation kit SQK-LSK110 (Oxford Nanopore Technologies, ONT, Oxford UK) following manufacturer’s guidelines. Libraries were purified by means of Agencourt AMPure XP magnetic beads (Beckman Coulter^™^) and sequenced employing flowcell flongle FLO-FLG001 version R10.4.1 adapted in a MinION Mk1C (ONT, Oxford UK) platform for 24 h. FastQ MinION files underwent quality control, trimming and reference assembly by Minimap2 plugin implemented in the software package Geneious Prime v. 2021.2.2 (Biomatters Ltd., Auckland, New Zealand).

### Sequence and phylogenetic analyses

Sequence analyses were performed by web-based tool BLAST^1^ applying default values to find homologous hits in the Genbank database. Sequence alignment was performed by MAFFT (31). The correct substitution model parameters for the phylogenetic analysis were obtained using “Find the best protein DNA/Protein Models” implemented in MEGA X version 10.0.5 software (32). Phylogenetic analysis was performed using the maximum likelihood (ML) method implemented in MEGAX version 10.0.5 software.

### GenBank sequence submission

The obtained sequence of CDV strain ITA/2022/badger/33340 were deposited in the Genbank database under accession number PQ584613.

## Results

Overall, out of 136 wild animals collected in Campania region in a period spanning from 2022 to 2024, 14 (10.3%) tested positive for CDV RNA by RT-qPCR (Table 1) with a cycle threshold (Ct) ranging from 17.8 to 42.5. CDV infections were observed in badgers (6/34, 17.6%), foxes (5/66, 7.6%), wolves (2/11, 18.2%) and martens (1/3, 33.3) while CDV was not retrieved from stone martens, porcupines and otters (Table 2). Six out 44 (13.6%) CDV positive cases (3 foxes, 2 badgers and 1 marten) were identified in 2022, 6/74 (8.1%) (3 badgers, 2 foxes and 1 wolf) in 2023, 2/18 (11.1%) (1 wolf and 1 badger) in 2024. CDV was unevenly identified in the lung, intestine, brain, spleen, liver, heart, muscle and kidney of the positive animals. The 14 CDV positive animals did not present co-infections with other analyzed pathogens. The 6 CDV-positive badgers were 3 males and 3 females and comprised 3 adults, 2 subadults and 1 young. The 5 CDV-positive foxes were 3 males and 2 females and included 3 adults, 1 subadult and 1 young. The 2 wolves were 1 male and 1 female and encompassed 1 adult and 1 subadult. The marten was an adult male. Out of 14 CDV-positive animals, the most frequent clinical observations at necroscopy were congested lungs and possibly also trachea in 4 (28.5%) animals followed by enteritis in 3 (21.4%) animals. The cause of death of the 14 CDV-positive animals were either trauma or infection (6/14, 42.8% for both), or gunshot and bite wounds (1/14, 7.1% for both). All the samples were tested for rabies virus and found to be negative.

**Table 1:** Information on animals infected by CDV: species, sex, age, organ CDV+, injuries, cause of death.

Year	ID	Province	Species	Sex	Age*	Organ CDV+	Injuries cause death	Cause of death
2022	28059	Naples	Fox	F	Ad	Intestine	Enteritis	Trauma
2022	31640	Salerno	Fox	M	Ad	Heart	Congested lungs	Trauma
2022	38077	Avellino	Marten	M	Ad	Lung, liver, kidney, heart, spleen	Not available	Trauma
2022	42191	Naples	Badger	F	Ad	Heart	Not available	Trauma
2022	42644	Salerno	Fox	F	Ad	Heart	Congested lungs	Infection
2022	108935	Salerno	Badger	F	Ad	Brain, lung, liver, kidney, intestine, heart, spleen	Marbled liver, Pneumonia	Infection
2023	6105	Salerno	Badger	M	SubAd	Brain, lung, liver, kidney, intestine, heart, spleen,	Enteritis, Congested lungs and trachea	Infection
2023	6164	Salerno	Badger	F	SubAd	Brain, lung, liver, kidney, intestine, heart, spleen,	Congested lungs and trachea	Trauma
2023	11970	Benevento	Wolf	F	SubAd	Spleen	Congested liver, hyperemic stomach, congested spleen, congested lungs and trachea	Gun
2023	31812	Benevento	Fox	M	SubAd	Brain, liver	Pulmonary edema, congested trachea	Infection
2023	33340	Salerno	Badger	M	J	Brain, lung, intestine, heart	Small splenomegaly? congested lungs and trachea	Infection
2023	57469	Caserta	Fox	M	J	Brain, lung, liver, intestine, heart, spleen	Enteritis, congested brain	Infection
2024	12478	Avellino	Wolf	M	Ad	Lung, liver, heart, spleen	Liver with hemorrhagic infarction, hyperemic stomach, splenomegaly, congested kidneys, congested lungs	Trauma
2024	14877	Salerno	Badger	M	Ad	Brain, lung, liver, kidney, intestine, heart, spleen	Congested lungs	Bite wounds

*Ad: Adult > 2 years; SubAd: Sub Adult (<2 years); J: young (6–12 months).

**Table 2:** Animal host species tested positive for CDV.

Host species	Years						Total	
	2022		2023		2024			
	CDV+ *n* (%)	Total	CDV+ *n* (%)	Total	CDV+ *n* (%)	Total	CDV+ *n* (%)	Total
Stone martens	0 (0.0)	0	0 (0.0)	4	0 (0.0)	0	0 (0.0)	4
Porcupines	0 (0.0)	2	0 (0.0)	10	0 (0.0)	0	0 (0.0)	12
Otters	0 (0.0)	0	0 (0.0)	5	0 (0.0)	1	0 (0.0)	6
Wolves	0 (0.0)	3	1 (20.0)	5	1 (33.3)	3	2 (18.2)	11
Martens	1 (50.0)	2	0 (0.0)	1	0 (0.0)	0	1 (33.3)	3
Badgers	2 (22.2)	9	3 (17.6)	17	1 (12.5)	8	6 (17.6)	34
Foxes	3 (10.7)	28	2 (6.2)	32	0 (0.0)	6	5 (7.6)	66
Total	6 (13.6)	44	6 (8.1)	74	2 (11.1)	18	14 (10.3)	136

CDV strain ITA/2022/badger/33340, retrieved from spleen of an adult male badger in 2022 (Ct = was subjected to full genome amplification and ONT sequencing protocol).

**Figure 1:**
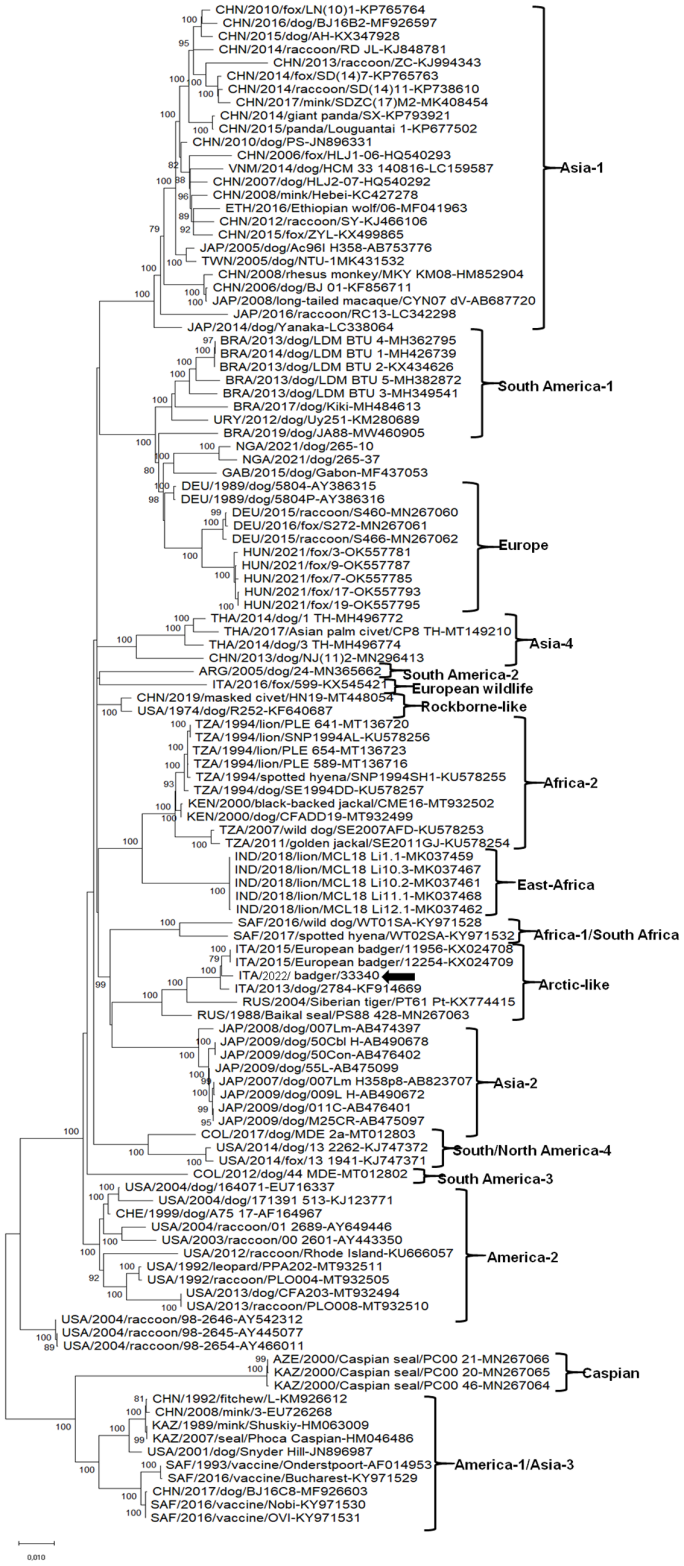
Phylogenetic tree obtained with the whole genome sequence of the badger CDV strains.

On full genome level, the CDV strain retrieved in this study displayed the highest nt identity (99.4%) to the isolate CDV11956/2015 (KX024708). The genomic sequence of the CDV strain ITA/2022/badger/33340 was aligned with cognate CDV sequences available in GenBank. Upon ML phylogenetic tree the CDV strain ITA/2022/badger/33340 segregated with other CDV strains included in the Arctic-like cluster (Figure 1).

## Discussion

The results of this study demonstrated the circulation of CDV in the wildlife in Southern Italy with infection rate equal to nearly 10%. No co-infections with other pathogens (i.e., CCoV, CAdVs type 1 and 2, CaHV-1, RVA and protoparvovirus carnivoran 1) were detected in the CDV-positive animals. In a previous report CDV infection rate reached 16% in tissue samples of foxes and badgers collected in Northern Italy (33). In this study, also, no other pathogens were detected in the animals that tested positive for distemper. In some cases, we can hypothesize, that the absence of other pathogens could also be related to the poor conditions of the carcasses.

About the rabies virus our territory has been declared free since 2013, checks are currently carried out only for constant monitoring, due to the presence of rabies in Eastern Europe. The complete genome sequence of the CDV strain, that we obtained from an adult male badger, phylogenetically characterized as an Arctic-like lineage, clustered along with other CDV strains retrieved from badger and dog from Italy.

The arctic lineages and the EuropeanWildlife have been mostly detected in Central and Southern Italy (14–16) while the Europe/South America-1 lineage is peculiarly spread in Northern Italy (13, 17, 34).

The Arctic CDV strains were first reported in animals of the Arctic ecosystem but this lineage appears common in European territories in both domestic and wildlife carnivores. In Italy, CDV strains belonging to the Arctic lieage were first reported in dogs in the early 21th century (35) and subsequently in the United States (36) and other European countries (2, 12, 37), raising questions on the origin of these unusual strains.

A large distemper epidemic, sustained by an Arctic-lineage (38), occurred in Italy during 2013 and involved primarily the Abruzzi region and neighboring regions, affecting foxes (*Vulpes vulpes*), badgers (*Meles meles*), beech martens (*Martes foina*) and European polecats (*Mustela putorius*) (14, 34).

Compared to other morbilliviruses, the CDV shows the highest genetic variability (39). The main factors fostering CDV spread, cross-species infection (40, 41) and increasing virulence (17, 42–45) are genetic variability, broad spectrum of hosts and the uncontrolled animal movements of stray and domestic dogs (2, 46–48) and other wild animals (49).

As previously observed in CDV infected animals, traumatic lesions appear a frequent cause of death in wild animals, probably due to vehicular collisions (33). In this study an equal number of CDV-positive animals dying from trauma and infections were observed.

A possible correlation between neurological disease due to either traumatic or infective etiology, and a delay in the animal’s response to external stimuli could be hypothesized (49).

Although the presence of specific clinical signs or anatomopathological lesions in live or dead animals could be related to CDV infection, confirmatory laboratory tests are needed to roule out other diseases. Molecular approaches could help monitoring CDV spread in susceptible wild animal hosts, particularly endangered species, thus supporting global animal welfare.

To date, the presence of CDV in Southern Italy has been described in both domestic dogs (2, 35, 46, 50) and wildlife (51). Our study contributes to extend the knowledge on the epidemiology of CDV in wildlife.

Continuous surveillance of CDV occurrence in wildlife will help depicting a better picture the ecology of CDV in domestic and wildlife animals and safeguarding endangered species.

## Data Availability

The obtained sequence of CDV strain ITA/2022/badger/33340 were deposited in the Genbank database under accession number PQ584613.

## Footnotes

^1^ https://blast.ncbi.nlm.nih.gov/blast.cgi?page_type=blastsearch
